# The American Prisoners in Dartmoor 1813-1815

**Published:** 1991-12

**Authors:** Peter Trafford


### The American Prisoners in Dartmoor 1813-1815

**Peter Trafford, MB, BS, DPM**

The United States Congress declared war on the United Kingdom on 18 June 1812; a negotiated peace was secured by the Treaty of Ghent which was signed on Christmas Eve 1814 and ratified on 20 March 1815. Britain had been at war with France for some thirty years and the dispute with America arose over the impressment of foreign nationals into the Royal Navy and over the right of search of American vessels trading with Europe.

In January 1812 Jas.Monroe, the U.S. Minister in London claimed that 6257 American citizens had been impressed into the R.N. during the previous ten years. It has been estimated that one eighth of seamen in the R.N. were foreigners and three quarters of the R.N. other-ranks were pressed men.

The numbers of soldiers and sailors killed in action in the Anglo-American war were: U.S. 1877 G.B. 3433.

The number of prisoners in Britain peaked to 72000 in 1814 and 6473 names are recorded in the reception registers of Dartmoor Prison; among these there were 229 deaths. Throughout the whole war only three British seamen are reported to have died in American hands.

Dartmoor Prison was built between the years 1806-9 to house French prisoners-of-war who until that time had been held mostly in temporary camps or in the hulks of old men-of-war lying in estuaries close to the main naval bases. The prison was the brainchild of Sir Thomas Tyrwhitt, secretary to the Prince of Wales and Lord Warden of the Stanneries of Devon and Cornwall; it was sited at a height of 1500 feet on North Hessary Tor, about fifteen miles from Plymouth, on ground granted by the Prince of Wales as Duke of Cornwall and Lord of the Forest of Dartmoor. The prison was hailed as "probably the finest thing of its kind and worthy of the humanity and renown of Great Britain" in 1811, but was later described by Sir George McGrath (Medical Superintendent there 1814-16) as "that great tomb of the living".

The prison was enclosed by an outer stone wall, one mile long and 14 feet high, surrounding a military road and an inner wall from which guards could command the enclosed area without mixing with the inmates. There were originally five main buildings but the French had completed two more by 1812: they were stone barns 60 feet long with 2 feet square unglazed windows -- no heating, and no chimneys. There were two stone floors with double tiers of hammocks slung between cast-iron pillars, and an upper wooden floor making a cock-loft under the roof. Primitive lean-to sheds outside, served as kitchens and latrines. The original intention had been to house some 1500 men in each prison but this number was frequently exceeded.

A plentiful supply of fresh water was available from the prison leat which still feeds into the reservoir outside the main gate of the prison; thence underground conduits led to each of the principal buildings.

Clothing and bedding were issued to all prisoners and appropriate entries made in the reception register. Many men arrived with practically no possessions though most had been give a hammock bed and a blanket before reaching Dartmoor. The standard issue suit was of yellow kersey bearing the initials T.O. front and back. (The Transport Office of the Admiralty was responsible for the care of prisoners-of-war). Periodical issues of replacement items are recorded e.g. hat, shirt, trousers, waistcoat and shoes.

Food appears to have been adequate though very dull by modern standards. Victualling was by contract and a local merchant signed a £3000 bond to fulfil his contract. Criminal convictions are recorded in 1812 and in 1814 on account of deficiencies in the bread deliveries. There was a daily allowance of 1½lbs. of bread and ½lb of beef (in the gross), with fish and potatoes instead of the beef two days a week. Local people furnished a daily market for fresh produce which could be purchased by those able to pay. In this respect the American prisoners were for some time at a considerable disadvantage as compared with the French. The latter were well established in all the paid working parties which often took them outside the prison, and some months passed before an agreement was reached between the U.S. Government and their London agent, Reuben G. Beasley, on the payment of a cash allowance to prisoners.

The prison staff numbered only 30 in all — headed by a Captain R.N. whose second-in-command was designated "Navigator"! The Surgeon and Assistant Surgeon had the help of a Matron and a Seamstress. The whole establishment was guarded by battalions of County Militia on a rota basis, the troops being changed every two months. To contain the French a garrision of 300-500 had been employed but when the more troublesome Americans arrived, this number was raised to above 1200 and artillery was added.

Medical supplies for the War Prisons came from the Apothecaries' 'Hall at Blackfriars, London, and were delivered by sea. Leeches were permitted to be purchased locally. One winter order included the following items: 300lbs Honey. One 36gall. barrel Lemon Juice. Sheepskins.

Two doctors were responsible for the medical care of the American prisoners. Dr. George Magrath (later to become Sir George, Inspector of H.M. Fleets and Hospitals) was a tall thin one-eyed Scot, conscientious in his duties and reported as showing "unwearying devotion and activity in checking the small-pox outbreak in 1815". Elsewhere it is stated that "he achieved great popularity although he made vaccination compulsory". In 1816, after their return home, ex-prisoners presented a testimonial to the President of the United States "demonstrating the regard in which the prisoners held Dr. Magrath and expressive of the high sense they entertained of his humane exertions and well directed skill in alleviating as far as possible the sufferings and maladies to which they were exposed in their place of durance". In contrast, Magrath's Scottish assistant McFarlane, was not popular and was described as a "rough inhuman brute".

Some of Sir George Magrath's comments (extracted from his report of 1816) are shown as an appendix to this paper.

The inclement weather conditions on the high moors are well known and undoubtedly contributed to the captives' discomfort; the winter of 1813-1814 is said to have been the coldest for fifty years. During the following winter a petition for permission to have fires was refused. Recorded comments on the severity of the weather lead one to believe that the French were more affected in this way than the Americans. Possibly the weather and the prevailing conditions on the moor itself discouraged escape attempts; certainly the presence of naval press gangs at all the major ports was a strong disincentive. It is believed that a score of Americans marched out of the prison in June 1814 with the final draft of French prisoners on their way to repatriation after the Anglo-French peace of May 1814 and before the escape of Napoleon from Elba led to a resumption of hostilities.

There was discontent and rising tension in the prison during the early months of 1815. Reuben G. Beasley, the U.S. Agent in London was never popular with his fellowcountrymen in Dartmoor and his communication with them was only through the Transport Board: one wonders how closely he was able to keep in touch with the United States government. The treaty of Ghent had been signed on Christmas Eve 1814 but three months later the prisoners had received no news of their repatriation. In fact, although the U.S. Government had granted no funds for the chartering of suitable vessels, Beasley had obtained transports which lay in the Downs off Plymouth for several weeks awaiting for a favourable wind to enter the port. On 25 March the prisoners staged a mock trial of Beasley in the presence of 5700 witnesses and convicted him, in his absence, of "depriving his countrymen of their lives by wanton and cruel deaths, by nakedness, starvation and exposure to pestilence". He was hanged in effigy on the top of no. 7 prison.

On 3 April there was a riot over shortage of bread; some escapes were made over the wall but a number of men returned the next day fearing the loss of their passage home. Serious rioting occurred three days later, but the exact cause remains doubtful. It culminated in confrontation with the raw Somerset Militia and shots were fired resulting in the deaths of ten Americans.

An official enquiry headed by Admiral Sir John Duckworth, Commander-in-Chief Plymouth and Major General Brown exonerated Captain Shortland of all blame, but the American account of the affair was so different that Lord Castlereagh, the Home Secretary, appointed an International Commission, with an American member, to investigate. The report of the Commission revealed little; it decided that there had been no real intention to escape or to mutiny, but that the behaviour of the prisoners was such that Captain Shortland was not to blame for believing that this was their intention. No justification was found for firing in the yards on unarmed prisoners. It failed to identify who gave the order to fire or to idendity the militiamen who fired without orders.

Depression and apathy followed — affecting both captors and captives alike; some escapes occurred — on one occasion eleven men got away in broad daylight without interference from the guards. The first draft of 242 liberated Americans marched out on 20 April 1815: a week later a party of 350 refused to be escorted to Plymouth by the Somerset Militia. All the Americans had gone by the end of the summer, the negroes from Prison 6 being the last to leave. Already in July there had been a new influx of 4000 French prisoners following the renewal of hostilities on the Continent — some of these had been taken at Waterloo.

The only documents I have been able to consult which give details of individual prisoners are the Reception Registers (4 volumes), and the death certificates. The former contain interesting details of personal appearance, scars and other distinguishing marks as well as information as to where captured and from what ship. The certified causes of death of 229 seamen are analysed on the slides; clearly there was a very serious outbreak of small-pox early in 1815 and I am surprised at the small number of bowel infections. Tuberculosis must have been rampant and I wonder how many of the acute cases were recorded simply as pneumonia rather than as phthisis. There is only one suicide — a 22 year old who "hung himself in no. 5 prison". Curiously this case is not among the three which were reported to the Coroner. The first American death occurred on 8 February 1814 and the last on 24 July 1815 — twenty year old William Meads, born in North Carolina and captured in the Privateer Snapdragon taken off Cuba. The oldest prisoner to die was a 62 year old who died from "anasarca" and the youngest a 12 year old ship's boy from the Privateer Harlequin taken off Halifax.

My talk is illustrated by slides showing some of the locations and features and documents mentioned; the documents are in the Public Record Office in London.

### APPENDIX
### SIR GEORGE McGRATH 1816

"The health of its incarcerated tenants in a general way equalled, if not surpassed, any war prison in England or Scotland. This might be considered an anomaly in sanitary history when we reflect how ungenially it might be supposed to act on southern constitutions, for it was not unusual, in the months of January and February for the thermometer to stand at 32 to 35 degrees below freezing, indicating cold almost too intense to support animal life. But the density of the congregated numbers in the prison created an artificial climate which counteracted the torpifying effect of the "Russian" climate without. Like most climates of extreme heat or cold, the newcomers required a "seasoning" to assimilate their constitution to its peculiarities, in the progress of which, indispositions incidental to low temperatures assailed them, and it was an everyday occurrence among the reprobate and incorrigible classes of prisoners who gambled away their clothing and rations, for individuals to be brought up to the receiving room in a state of suspended animation from which they were usually resuscitated by the process resorted to in like circumstances in frigid regions. I believe one death only took place during my sojourn at Dartmoor from torpor induced by cold, and the profligate part of the French were the only sufferers. As soon as the system became acclimated to the region in which they lived, health was seldom disturbed.

During my service there, malignant measles, and smallpox were imported from other contaminated sources. These diseases attained to great virulence among the Americans chiefly arising from habits of indulgence, from the ample pecuniary resources they possessed, and the facilities of obtaining spirits and sumptuous articles of diet from the market-people, which no vigilance on the part of the authorities could suppress or obviate. The latter disease degenerated into an exasperated species of peri-pneumonia accompanied by low typhoid symptoms which became very unmanageable and destructive. Independently of these contagious epidemics the depot may be said to have been surprisingly healthy.

I posses no register of the conditions of health or disease obtaining in other war prisons so as to enable me to draw an accurate parallel, but Dartmoor was generally considered equal if not superior to any depot where the same number of men were confined in so narrow a compass; but is must be borne in mind that after the closing of Mill Bay prison (that is just outside Plymouth) Dartmoor received men from the Colonies, long shut-up in transports and often landed with the seeds of infection generated among them, and predisposed by privation and a vitiated atmosphere, to disease, while none were sent to prisons in the interior, but men selected on purpose in perfect health".

**Table 1**

Total Deaths of American Prisoners in Dartmoor Prison

| 1813 | | 1814 | | 1815 | |
|---|---|---|---|---|---|
| April | 1 | January | 6 | January | 44 |
| July | 1 | February | 3 | February | 34 |
| October | 2 | March | 5 | March | 38 |
| November | 2 | April | 2 | April | 25 |
| December | 1 | May | 1 | May | 8 |
| | | June | 1 | June | 10 |
| | | July | 3 | July | 2 |
| | | August | 2 | | |
| | | September | 5 | | |
| | | October | 15 | | |
| | | November | 5 | | |
| | | December | 13 | | |

**Table 2**

**Main Causes of Death (Total 229)**

| | |
|---|---|
| Smallpox | 74 |
| Pneumonia | 52 + 3 "Pulmonic complaint" |
| Phthisis | 34 + 4 "Tabes mesenterica, haemoptysis" |
| Bowel infections | 15 (enteritis, dysentary & diarrhoea) |
| Killed | 10 (including 7 'shot in prison yard' on 6/4/15) |
| Suicide | 1 |

